# Depression and anxiety in female refugees from East Africa and the Middle East displaced to Germany: cross-sectional results of the female refugee study, taking sociodemographic and migration-related factors into account

**DOI:** 10.3389/fpsyt.2023.1303009

**Published:** 2024-01-04

**Authors:** Helena Katharina Bohland, Renate Kimbel, Peter Kegel, Pavel Dietz, Clemens Koestner, Stephan Letzel, Christine Kurmeyer, Jenny Jesuthasan, Meryam Schouler-Ocak, Ulrike Zier

**Affiliations:** ^1^Institute of Occupational, Social and Environmental Medicine, University Medical Centre, Johannes Gutenberg University Mainz, Mainz, Germany; ^2^Women and equal opportunities office, Charité – Universitätsmedizin Berlin, Berlin, Germany; ^3^Psychiatric University Clinic of Charité at St. Hedwig Hospital, Berlin, Germany

**Keywords:** depression, anxiety, mental health, female refugee, traumatic event

## Abstract

At the end of 2022, 108.4 million people around the world were forcibly displaced, the highest number ever recorded. Of these, 50% were women. Despite this situation, little is known about the mental health of female refugees. The first aim of this study was to examine the prevalence of depression and anxiety symptoms among female refugees in Germany. The second aim was to examine which sociodemographic and migration-related variables have an impact on refugees’ mental health, and the third aim was to assess the potential predictors of their mental health. A sample of 92 female refugees from East Africa and the Middle East living in Germany were interviewed. Symptoms of depression and anxiety were assessed using the Hopkins Symptom Checklist (HSCL-25). The experience of potentially traumatic events (PTEs) was assessed using the Posttraumatic Diagnostic Scale (PDS) and the Harvard Trauma Questionnaire (HTQ). In our sample of female refugees, 65.2% reported symptoms of depression, and 60.9% reported symptoms of anxiety. Symptoms of depression or anxiety were associated with being from the Middle East, having a higher level of education, and reporting more PTEs. The multiple regression model for anxiety was able to explain 32.4% of the variance in anxiety symptoms. The findings highlight the high burden of mental health problems that female refugees bear. The identified predictors of depressive and anxiety symptoms should sensitize medical and refugee professionals to identify vulnerable individuals and groups, refer them to appropriate psychological treatment, and, where possible, modify the identified predictors.

## Introduction

In 2022, the number of forcibly displaced persons in the world was the highest on record (1). The United Nations High Commissioner for Refugees (UNHCR) has estimated that there are 108.4 million forcibly displaced people worldwide, of whom 35.3 million are refugees (1). In 2016, when the interviews for this study began, more than 720,000 asylum applications were registered in Germany (2), the highest number ever recorded in Germany. Women accounted for 38% of all asylum applications in Germany in 2016 (2). The five most represented countries of origin that year were Syria, Afghanistan, Iraq, Iran, and Eritrea. At the time of the interviews for this study, the countries with the best prospects of obtaining a right of residence were Afghanistan, Iraq, Iran, Eritrea, Somalia, and Syria. It is important to know about the experiences of refugees and the measures taken in 2016 that can help people seeking protection in Germany today. However, the refugee population today is different in terms of country of origin, the circumstances of flight, and the reception conditions in Germany (2, 3). In 2022, Germany recorded the highest increase of people seeking protection^1^ in the country since records have been kept (3). Approximately 1.1 million refugees from Ukraine have sought protection in Germany as a result of the international conflict between Russia and Ukraine (3, 4). In 2022 also, around 47.0% of people seeking protection in Germany were female, and among Ukrainian refugees, up to 64% were girls and women^2^ (3). In response to the influx of refugees from Ukraine, the EU invoked the Temporary Protection Directive (5), according to which refugees from that country are granted a residence permit for one to three years without any bureaucratic application procedure (5). This grants them, for example, the right to stay in a private flat instead of a collective accommodation and access to statutory health insurance instead of emergency health insurance (6, 7). Therefore, a comparison between the refugee population in 2016 and today is only possible to a limited extent.

The UNHCR reports that women are a vulnerable group within the refugee population (8, 9). They experience more gender-based and sexual violence than men (10) and thus require special attention to address their needs (11). Research on refugees’ mental health shows that female refugees have more mental health problems than male refugees (9, 10, 12, 13). Mental health disorders, such as depression and anxiety, are also more common among female refugees than among male refugees (13). A systematic review by Morina et al. (14) found that most studies in refugee populations focus on posttraumatic stress disorder (PTSD), depression and anxiety. There are few studies examining other mental health disorders. Morina et al. (14) point out, that the prevalence of all mental disorders must be higher in the refugee population due to the difficult circumstances during and after flight. Also chronic mental health problems may get worse under the lack of treatment (14). It is clear that the presence of mental health problems makes integration into the host country more difficult (15). It remains to be seen, however, what impact mental health problems in refugee mothers have on the integration of their children.

At the same time, a review by Blackmore et al. found great heterogeneity in the reported prevalence of mental health problems, such as symptoms of depression and anxiety, in refugee populations and a lack of data on gender-specific mental health problems in these populations (16). Comparison with other studies shows that the prevalence of symptoms of depression or anxiety in the female refugee population is rarely reported (10, 17). Studies on the mental health of male and female refugees often consist mainly of male refugees, do not report their findings by gender, age, or ethnicity, and report heterogeneous prevalence rates for symptoms of depression and anxiety (10, 17).

More research is thus needed on the prevalence of symptoms of depression and anxiety in female refugee populations. The recent influx of Ukrainian refugees to Germany, most of whom are women (64.0%), underlines the importance and timeliness of this issue (3).

The refugee population in Germany and around the world is heterogeneous in terms of, for example, country of origin, route of flight, opportunities for residency in, and reception in the host country. It is therefore important to understand which sociodemographic and migration-related factors are associated with the mental health of refugees.

Reviews indicate that the association between mental health and sociodemographic and migration-related variables has not been well studied (10, 17–19). Only a few reviews have examined this association (10, 17–19), and the reported results are heterogeneous. Studies have shown that an older age (9, 10, 20–22) and having experienced more PTEs (21, 23–25) are associated with mental health problems. In their review, Mesa-Vieira et al. (10) reported that migrants with previous exposure to violence were likely to have poor mental health. Sundquist et al. (26) reported that economic difficulties in the host country appeared to be an even greater factor in poor mental health than exposure to violence prior to migration. Mesa-Vieira et al. (10) found that lower income levels in the host country and, in contrast to the above-mentioned studies, a younger age were associated with poor mental health (10). Lindert et al. (17) found in their review that a high level of income in the host country may be associated with better mental health in labor migrants, but not in refugees. Often, the reviews include refugees as well as migrants and labor migrants (10, 17); therefore, subgroup analyses by gender are not possible (17).

Regarding the relationship between the country of origin and mental health among refugees, the results are heterogeneous. Some studies have found significant differences in symptoms of depression and anxiety according to country of origin (23, 27, 28), while others have found no association between country of origin and mental health (24). Few studies have examined the association between the level of education and mental health in refugee populations. However, in those that do, the results are heterogeneous. A positive association between the level of education and symptoms of depression and anxiety (29), a negative association (9), and no association (22) have been reported. In addition, there is a lack of research on post-migration factors that may be associated with refugees’ mental health, and current stressors in the lives of refugees should also be considered (18).

To address the above-mentioned research gaps, the first aim of this study was to investigate the prevalence of symptoms of depression and anxiety in female refugees. The second aim was to examine the effects of sociodemographic and migration-related variables on mental health. Literature research was conducted to examine the sociodemographic and migration-related variables relevant to refugees’ mental health. The third aim was to assess the potential predictors of mental health among female refugees.

## Method

The present study was part of a larger research project, the Female Refugee Study (FRS) (30). This multicenter study was conducted in 2016 and 2017 in six German federal states and was funded by the State Commissioner for Migration, Refugees and Integration of the German government. The present study examines data from the Rhineland-Palatinate study center that we collected for the FRS. Ethics committee approval was obtained on September 19, 2016, and the study is registered in the Ethics Committee Register of the Rhineland-Palatinate Medical Association under the number 837.316.16 (10635). Inclusion criteria were female gender, age 18 years and older, cognitive capacity to be interviewed (verbally or in writing), ability to speak and understand one of the provided study languages (Arabic, Dari/Farsi, Somali, Tigrinya), living in a refugee reception center, and beingfrom Afghanistan, Eritrea, Iran, Somalia, and Syria. At that time, refugees from these countries had a high chance of being granted asylum in Germany. Recruitment took place in randomly selected refugee reception centers in Rhineland-Palatinate. The owner and operator of the reception centers for refugees, granted permission for the study. Female refugees were recruited through a 1.5-h information sessions at refugee reception centers. Each woman interested in participating in the study was interviewed privately after at least a 1-day interval. Informed consent was obtained either in writing or verbally. The interviews were conducted by trained bilingual female interviewers either as a guided questionnaire assessment or as a structured interview. Interviewers did not insist to answering to minimize the risk of retraumatizing. The questionnaire consisted of 71 questions, including psychometric instruments for trauma experience, quality of life, and symptoms of depression and anxiety. The questionnaire was translated (forward-backward translation) and adapted for ethical differences. Sociodemographic and migration-related data were assessed using investigator-related questions. As the dependent variables, the symptoms of depression and anxiety were assessed using the Hopkins Symptom Checklist (HSCL) (31–33), an instrument commonly used with refugee populations (34). The HSCL includes 25 symptoms of depression and anxiety, the presence and severity of which over the past 7 days are rated on a 4-point Likert scale (1 = not at all to 4 = very much). The HSCL symptom score is the mean of the responses. A cut-off value of ≥1.75 is commonly used to differentiate clinically relevant symptoms of depression and anxiety (35). The HSCL does not provide a clinical diagnosis; however, it does allow the severity of symptoms of depression and anxiety to be assessed and compared using symptom scores.

The independent variables are age in years, level of education as years of schooling, number of children, country of origin, stay in Germany (in months), marital status (single/partnership, cohabitation/partnership, separation), separation from children (yes/no), number of PTEs, experience of physical, psychological, or sexual violence (yes/no), distressing experiences in Germany (yes/no), and satisfaction with living conditions in Germany (rather satisfied/rather not satisfied). Regarding the assessment of the number of PTEs, the Posttraumatic Diagnostic Scale (PDS) (36, 36) and the Harvard Trauma Questionnaire (HTQ) (37) were used.

Descriptive analyses of sociodemographic and migration-related variables were then performed. The prevalence of symptoms of depression and anxiety was calculated using the common cut-off (≥ 1.75) for the HSCL (35). Exploratory analyses of the data were then performed. Bivariate statistics were used to analyze the association between symptoms of depression and anxiety and sociodemographic and migration-related variables. For the bivariate analysis, the independent variables were transformed into categorical variables when necessary. Due to the non-parametric data, the Mann–Whitney *U*-test and the Kruskal-Wallis H-test were calculated, with a value of p of <0.05 considered significant. In the next step, the variables indicating an association with symptoms of either depression or anxiety in the bivariate analyses were entered into logistic regression models, with a value of *p* of <0.05 considered significant. Except for country of origin, the continuous version of the variables was used to calculate the regression models. The variables included in the logistic regression models were age (years), level of education (years), number of children, country of origin (Middle East/East Africa), and number of PTEs. Bivariate analysis indicated an association between the categorical variable of experience of physical, psychological, or sexual violence and symptoms of depression and anxiety. For precision and statistical reasons, instead of the categorical variable experience of physical, psychological, or sexual violence, we included the continuous variable number of PTEs in our regression models. Diagnosis of the calculated regression models was then performed. All statistics were performed using SPSS Statistics Version 23 (IBM, Armonk, NY).

## Results

At the time of the study, 332 female refugees in Rhineland-Palatinate met the inclusion criteria. 92 female refugees participated in our study. Most of the female refugees interviewed were from Afghanistan (34.8%) and Syria (32.6%). They had been in Germany for between 2 and 27 months (median 12 months). The female refugees interviewed were mostly young women (median 29 years, mean 31.7 years). The study population differed according to their origin. Refugees from the Middle East were, on average, 5 years older than refugees from East Africa. Female refugees from Iran reported the highest level of education, followed by refugees from Eritrea and Syria. The lowest level of education was reported by female refugees from Somalia. Most female refugees interviewed reported being in a relationship (54.3%). Refugees from the Middle East were likelier to report having children (82.0%) than refugees from East Africa (56.0%). Of the women interviewed, 19.6% reported having been separated from their children.

The number of reported PTEs varied by country of origin. Refugees from Africa reported, on average, a higher median number of different PTEs (Eritrea: 12.0; Somalia: 12.5) than refugees from the Middle East (Afghanistan: 7.5; Iran: 6.0; Syria: 3.5). Female refugees in a partnership reported fewer PTEs than did women without a partnership. Female refugees without a partnership were likely to report six or more different PTEs (81.0%). Among the female refugees interviewed in a partnership, 46.0% reported having experienced six or more PTEs (see Table 1). The five most frequent reported PTEs were “forced separation from family members” (58.7%), “lack of shelter” (57.6%), “being close to death” (54.3%), “lack of food or water” (51.1%) and “ill-health without access to medical care” (46.7%).

**Table 1:** Sociodemographic and migration-related data of the study population (*n* = 92).

	Study population *n* = 92 (100%) *n* (%)	Middle East *n* = 67 (72.8%) *n* (%)	East Africa *n* = 25 (27.2%) *n* (%)
Age in years			
[*Median; IQR*] 18–22 23–27 28–32 33–37 ≥38	[29.0; 12.0] 15 (16.3) 20 (21.7) 22 (23.9) 13 (14.1) 22 (22.9)	[32.0; 12.0] 8 (11.9) 13 (19.4) 15 (22.4) 10 (14.9) 21 (31.3)	[27.0; 8.0] 7 (28.0) 7 (28.0) 7 (28.0) 3 (12.0) 1 (4.0)
Level of education in years			
[*Median; IQR*] 0 1–6 7–9 ≥ 10 n.a.	[7.0; 9.8] 20 (21.7) 19 (20.7) 18 (19.6) 27 (29.3) 8 (8.7)	[7.0; 12.0] 16 (23.9) 11 (16.4) 11 (16.4) 21 (31.3) 8 (11.9)	[7.0; 7.0] 4 (16.0) 8 (32.0) 7 (28.0) 6 (24.0) –
Country of origin			
Afghanistan Eritrea Iran Somalia Syria	32 13 5 12 30	32 5 30	13 12
Stay in Germany in months			
[*Median; IQR*] ≤6 7–12 ≥ 13 n.a.	[2.0; 6.0] 15 (16.3) 25 (27.2) 36 (39.1) 16 (17.4)	[12.0; 4.0] 8 (11.9) 23 (34.3) 28 (41.8) 8 (11.9)	[11.0; 12.5] 7 (28.0) 2 (8.0) 8 (32.0) 8 (32.0)
Marital status			
Single Partnership, cohabitation Partnership, separated	18 (19.6) 50 (54.3) 24 (26.1)	7 (10.4) 45 (67.2) 15 (22.4)	11 (44.0) 5 (20.0) 9 (36.0)
Number of children			
[*Median; IQR*] 0 1–3 ≥4	[2.00; 2.8] 23 (25.0) 48 (52.2) 21 (22.8)	[3.00; 3.0] 12 (17.9) 34 (50.7) 21 (31.3)	[1.00; 2.0] 11 (44.0) 14 (56.0) –
Separation from children			
Separated Not separated/no children n.a.	18 (19.6) 67 (72.8) 7 (7.6)	13 (19.4) 47 (70.1) 7 (10.4)	5 (20.0) 20 (80.0) –
Number of PTEs experienced			
[*Median; IQR*] 0 1–5 6–10 ≥ 11 n.a.	[8.0; 10.0] 9 (9.8) 24 (26.1) 27 (29.3) 30 (32.6) 2 (2.2)	[6.0; 8.0] 9 (13.4) 23 (34.3) 19 (28.4) 15 (22.4) 1 (1.5)	[12.0; 5.0] – 1 (4.0) 8 (32.0) 15 (60.0) 1 (4.0)
Experience of physical, psychological, or sexual violence			
Yes No n.a.	74 (80.4) 16 (17.4) 2 (2.2)	50 (74.6) 16 (23.9) 1 (1.5)	24 (96.0) – 1 (4.0)
Distressing experience in Germany			
Yes No n.a.	27 (29.3) 54 (58.7) 11 (12.0)	15 (22.4) 45 (67.2) 7 (10.4)	12 (48.0) 9 (36.0) 4 (16.0)
Satisfaction with living conditions in Germany			
Rather satisfied Rather not satisfied n.a.	40 (43.5) 50 (54.4) 2 (2.2)	25 (37.3) 40 (59.7) 2 (3.0)	15 (60.0) 10 (40.0) –

n.a., not applicable; IQR, interquartile range.

Analyses of the HSCL revealed symptoms of depression in 65.2% of the female refugees interviewed and symptoms of anxiety in 60.9% (n = 88). Symptoms of depression and anxiety were more common in Middle Eastern female refugees than in East African female refugees. The HSCL symptom scores differed significantly according to country of origin (depression score median Middle East 2.26, median East Africa 1.86, *p* = 0.018; anxiety score median Middle East 2.40, East Africa 1.60, *p* = 0.001) (see Table 2).

**Table 2:** Symptoms of depression and anxiety.

	Depression Scale *n* = 88 (n/%)	Anxiety Scale *n* = 88 (n/%)
Median (IQR)	2.17 (1.02)	2.05 (1.30)
Range	1.00–3.87	1.00–3.80
Symptom score ≥ 1.75	60 (65.2)	56 (60.9)
Symptom score < 1.75	28 (30.4)	32 (34.8)
n.a.	4 (4.3)	4 (4.3)

Results of the HSCL. n.a., not applicable; IQR, interquartile range.

Bivariate analyses showed that symptoms of depression were significantly associated with being from the Middle East (Mann–Whitney U-Test, value of *p* <0.05), reported exposure to violence (Mann–Whitney U-Test, value of *p* <0.05), and the number of children (Kruskal-Wallis H-Test, value of *p* <0.05). Symptoms of anxiety were significantly associated with an older age (Kruskal-Wallis H-Test, value of *p* <0.05), being from the Middle East (Mann–Whitney U-Test, value of *p* <0.01), a high level of education (Kruskal-Wallis H-Test, value of p <0.05), a high number of children (Kruskal-Wallis H-Test, value of p <0.05), and reported exposure to violence (Mann–Whitney U-Test, value of p <0.05) (see Table 3).

**Table 3:** Association between sociodemographic and migration-related data and symptoms of depression and anxiety, bivariate analyses.

Variables	Depression Scale *n* = 88 *Median (IQR)*	Anxiety Scale *n* = 88 *Median (IQR)*
Age in years *(K-W)*		*
18–22 23–27 28–32 33–37 ≥38	1.87 (1.51) 2.03 (0.82) 2.21 (1.39) 2.14 (1.18) 2.35 (1.15)	1.60 (1.50) 1.80 (0.88) 2.10 (1.45) 2.56 (1.35) 2.75 (1.13)
Origin *(M-W)*	*	**
Middle East East Africa	2.26 (1.07) 1.86 (0.74)	2,40 (1,30) 1,60 (0,68)
Level of education in years *(K-W)*		*
0 1–6 7–9 ≥ 10 n.a.	2.14 (1.08) 1.97 (0.95) 2.27 (1.14) 2.27 (1.20) 1.73 (1.17)	1.90 (1.62) 1.90 (1.00) 2.15 (1.58) 2.70 (1.23) 2.20 (1.30)
Stay in Germany in months (*K-W*)		
≤6 7–12 ≥ 13 n.a.	2.33 (0.83) 2.37 (0.98) 1.87 (1.13) 1.87 (1.47)	2.00 (1.60) 2.27 (1.30) 2.00 (1.17) 1.90 (1.49)
Marital status (*K-W*)		
Single Partnership, cohabitation Partnership, separated	1.86 (0.65) 2.20 (1.17) 2.30 (1.03)	1.89 (1.14) 2.10 (1.50) 2.25 (1.23)
Number of children *(K-W)*	*	*
0 1 2 3 4 5 6 7 (*n* = 1) 8 (*n* = 1)	1.87 (0.73) 1.60 (0.93) 1.70 (0.73) 2.47 (0.59) 2.63 (0.55) 1.27 (2.07) 2.33 (−) 1.40 (−) 1.80 (−)	1.89 (1.10) 1,80 (0.80) 1.63 (1.23) 2.70 (1.10) 2.80 (0.78) 1.60 (1.49) 2.33 (–) 3.20 (–) 1.50 (–)
Separation from children *(M-W)*		
Separated Not separated/no children n.a.	2.33 (1.00) 2.10 (1.10) 2.13 (0.91)	2.50 (1.29) 2.00 (1.23) 2.85 (1.40)
Number of PTEs experienced (*K-W*)		
0 1–5 6–10 ≥ 11 n.a.	1.87 (0.87) 2.21 (1.35) 1.93 (1.41) 2.30 (0.81) 1.60 (−)	1.56 (0.59) 2.15 (1.59) 2.00 (1.35) 2.50 (1.35) 1.40 (−)
Experience of physical, psychological, or sexual violence *(M-W)*	*	*
Yes No n.a.	2.24 (1.08) 1.80 (0.92) 1.60 (−)	2.27 (1.30) 1.58 (0.78) 1.40 (−)
Distressing experience in Germany *(M-W)*		
Yes No n.a.	1.93 (1.33) 2.14 (1.03) 2.55 (1.18)	2.20 (1.32) 2.05 (1.28) 2.30 (1.65)
Satisfaction with living conditions in Germany *(M-W)*		
Rather satisfied Rather not satisfied n.a.	2.10 (0.99) 2.21 (1.16) 1.69 (−)	2.05 (1.31) 2.10 (1.35) 1.56 (−)

*p < 0.05; **p < 0.01; IQR, interquartile range; n.a., not applicable. K-W, value of *p* computed with Kruskal–Wallis *H*-Test. M-W, value of *p* computed with Mann–Whitney *U*-Test.

The regression model for anxiety symptoms, including the variables of age, level of education, number of children, country of origin, and number of PTEs, explained more than 32.4% (*p* < 0.001) of the variance in anxiety symptoms. Origin (standardized beta coefficient 0.301, *p* = 0.007) and the number of PTEs (standardized beta coefficient 0.397, *p* = 0.002) explained the most variance in anxiety symptoms. Besides country of origin and the number of PTEs, the variable of level of education added a significant explanation to the variance in symptoms of anxiety in the model.

The regression model for symptoms of depression, including the variables of age, education level, number of children, country of origin, and number of PTEs, showed no indication of a significant association (see Table 4).

**Table 4:** Multiple linear regression models of sociodemographic variables and number of PTEs and symptoms of depression and anxiety.

	Model depression	Model anxiety
	Standardized beta-coefficients (value of *p*)	
Age	0.053 (0.741)	0.254 (0.071)
Education	0.096 (0.412)	0.235 (0.023) *
Number of children	0.039 (0.824)	−0.054 (0.726)
Origin (Middle East/East Africa)	0.280 (0.054)	0.397 (0.002) **
Number of PTEs	0.187 (0.139)	0.301 (0.007) **
Fit of the model – *R*^2^ (value of *p*)	0.110 (0.122)	0.324 (< 0.001) **

*p < 0.05; **p < 0.01.

Data were missing by 4.3% in the symptom scales and 0.0–8.7% in the sociodemographic and migration-related values. Missing data were excluded.

## Discussion

As far as we know, the FRS was the first study of its kind in Germany. The cross-sectional data from the Rhineland-Palatinate discussed in the present study constitute a unique database of the mental health of female refugees. Our aim was to interview all female refugees in the Rhineland-Palatinate who met the inclusion criteria of the FRS. Almost one-third of this population was interviewed, so the data provided good insight into the mental health of female refugees in the Rhineland-Palatinate.

In our study population, the prevalence of symptoms of depression was 65.2%, and it was 60.9% for symptoms of anxiety. These prevalence rates are high, and given that they represent the burden of only two mental disorders among other possible disorders from which the female refugees interviewed may have suffered, the reported prevalence rates are worrying.

A comparison with the general population shows the expected higher burden of mental health disorders among female refugees (22, 38). The mean symptom score for depression (HSCL) in women in the general population without a clinical diagnosis is 1.30 (SD 0.42), and for anxiety, it is 1.29 (SD 0.34) (31). The median symptom score for depression and anxiety found in this study in the surveyed female refugee population is much higher, with a symptom score of 2.17 (IQR 1.02) for depression and 2.10 (IQR 1.34) for anxiety. These symptom scores in our sample are unexpectedly high compared with women in the general population diagnosed with a depressive or anxiety disorder. The mean symptom score for depression in women in the general population with a clinical diagnosis of a depressive disorder is 1.61 (SD 0.62), and for anxiety it is 1.60 (SD 0.47) (39). This highlights the exceptionally high burden of symptoms of depression and anxiety experienced by female refugees. In addition, it underscores the need for mental health treatment for this population. At the same time, medical care for refugees in Germany is limited in the first months after arrival to the treatment of acute illnesses or pain (40). And even when medical care is available, there are many other barriers to adequate health care for refugees, such as language barriers, lack of knowledge of structures and where to go for help, lack of trust in the healthcare system, or inability to talk about intimate feelings (41). And even when refugees do receive medical care, there is a risk of under-diagnosis due to cultural differences in the presentation of symptoms (41). It is clear that mental disorders in female refugees make integration into Germany and learning the host language difficult, if not impossible, for them (15). The impact on their children must also be considered. Three-quarters of the surveyed refugees reported having children, and caring for children is surely even more difficult with the burden of mental illness.

Country of origin was associated with symptoms of depression or anxiety. The symptoms of both depression and anxiety were higher among female refugees from the Middle East. These findings are consistent with the results of a review by Blackmore et al. of populations of refugees and asylum seekers (16). The differences in the symptoms of depression and anxiety by country of origin may be explained by the loss of socioeconomic status following flight. Female refugees from the Middle East may experience a greater loss of socioeconomic status after their arrival in Germany than refugees from East Africa. Another reason may be culturally different strategies for coping with mental health problems. The presentation of symptoms of depression and anxiety is known to vary from culture to culture, which can lead to misdiagnosis (41).

The experience of physical, psychological, or sexual violence was associated with symptoms of depression and anxiety in our sample. The number of PTEs showed a significant association with the variance of anxiety symptoms, just below level of education. This is consistent with other studies showing a strong association between the number of PTEs and the manifestation of mental disorders (21, 23–25). The experience of PTEs in refugees, home country or during their flight, e.g., being close to death, lack of food, water and shelter, increases the risk for a wide spectrum of mental diseases, e.g., PTSD, depression, anxiety and somatization.

Unexpectedly, there was no evidence of an association between the symptoms of depression and anxiety and age in our sample. The multivariate regression model showed that age was not significantly associated with symptoms of depression and anxiety when controlling for level of education, number of PTEs, and country of origin. In contrast are the findings pertaining to the German general population and other refugee populations. In the general German population, a representative study from 2009 to 2012 showed that the prevalence of depression decreases with age: 15.6% of younger women, 11.0% of middle-aged women, and 5.0% of older women suffered from a depressive disorder (42). Other studies of refugee populations report that older age is associated with more symptoms of mental health disorders (9, 10, 20–22). Our sample of female refugees consisted mostly of young female refugees (median age 29 years), making it difficult to measure associations in higher age groups. Perhaps the lack of association between age and symptoms of depression and anxiety is due to our study population. On the other hand, our findings suggest that the number of PTEs experienced, the country of origin, and the level of education outweigh the influence of age on young female refugees.

Surprising in our results was the association between educational level and symptoms of depression and anxiety. A high level of education was significantly associated with high anxiety symptom scores. Symptoms of depression were likelier to be reported by female refugees with no or high levels of education than by female refugees with low to medium levels of education. These findings contrast with those of the general German population, where higher levels of education are associated with better mental health (43). Other studies of refugee populations have reported heterogeneous findings on the relationship between educational attainment and mental health (9, 22, 29). It should be kept in mind that higher levels of education may lead to a higher socioeconomic status in the home country and an even greater loss of socioeconomic status in the host country. This, in turn, is associated with symptoms of depression and anxiety, as Blackmore et al. found in their review (16).

Another unexpected finding was that the number of children was not associated with symptoms of depression or anxiety. In the regression models, no variance was explained by the number of children. Caring for children involves taking responsibility for others, a more difficult flight, and perhaps separation from one’s children. There is less time to nurture oneself, to learn a new language, and to get to know the host culture. All of this should lead to more stress for women with children than for those without children. However, in our sample, other factors seemed to be more important. Another explanation could be that the positive effect of having children and the motivation to give them a better future outweigh the burden of parenthood.

Only the regression model for symptoms of anxiety showed a significant explained variance of 32.4% (value of *p* <0.001). The explained variance is small, as is common in models predicting human behavior (44). Nevertheless, our model could explain only 32.4% of the variance in the symptoms of anxiety. The variables of level of education, number of PTEs experienced, and country of origin (East Africa/Middle East) added a significant explanation to the variance of symptoms of anxiety in our sample. This suggests that other factors that were not analyzed could have played a more important role in the variance in symptoms of anxiety in our sample.

The main limitation of this study is the small sample size (*n* = 92), which included only female refugees from the Rhineland-Palatinate study center. Due to the small sample size, the variables examined and the associations found must be interpreted carefully. Another limitation is the relatively high number of variables analyzed compared to the small sample size. Therefore, all significant results should be interpreted with caution.

Regarding other limitations, it must be kept in mind that the instrument used to assess psychological symptoms, although often used in research with refugee populations, has not been validated for Middle Eastern and African cultures (33, 45, 46). It is possible that the burden of depression and anxiety symptoms was over- or underestimated in our analyses. Further research and validation studies are needed to accurately measure the symptoms of depression and anxiety across cultures. The additional assessment of symptoms of posttraumatic stress disorder, would have provided a better picture of the mental health problems experienced by refugee women with high levels of PTEs. Additional Fazel et al. (47) could show in their review about the prevalence of serious mental disorders in refugees a high comorbidity of PTSD and major depression and vice versa. The National Comorbidity Survey in the United States found that half of the women diagnosed with PTSD were also diagnosed with major depression (48). There are two important explanations, as suggested by Flory and Yehuda (49), either these two disorders have many overlapping symptoms, or the combination of PTSD and major depression is a subtype of one disorder. Flory and Yehuda therefore state that the question should not be, whether there is a comorbidity of PTSD, but which comorbidity is present (49). This underscores the urgent need to assess symptoms of PTSD and depression together. For future research, we recommend assessing symptoms of posttraumatic stress disorder in refugee populations.

Missing data were low in our sample, with 4.3% missing values on the symptom scales and 0.0–8.7% missing values on the sociodemographic and migration-related measures. Nevertheless, this may have led to an over- or underestimation of psychological symptoms, and some associations with the assessed factors may have remained hidden.

Female refugees from Ukraine are currently the largest group of female refugees coming to Germany (3). Like other refugees, they are likely to suffer from mental disorders (50, 51). A study of Ukrainian refugees in Germany found a high prevalence of mental health disorders which is comparable to the prevalence in other refugee populations, with almost half of the sample reporting symptoms of depression and more than half reporting symptoms of anxiety (50). This is a remarkably high prevalence, considering that Ukrainian refugees currently have easier access to residence permits in Germany without any bureaucratic procedures (5). In addition, they are allowed to stay in private apartments instead of collective accommodations and have access to statutory health insurance rather than emergency health insurance (6, 7). Buchcik et al. (50) suggest that the war in the home country and migration-related experiences, such as leaving male relatives behind and uncertainty about the future, outweigh the easier reception conditions that Ukrainian refugees currently have in Germany.

Overall, refugee women are a vulnerable group. Female refugees from East Africa and the Middle East who were interviewed in this study had a high prevalence of symptoms of depression and anxiety. Experiencing symptoms of a mental disorder can lead to a high burden of disease and difficulties in integration into the host country. For healthy integration, it is necessary to identify the symptoms of mental disorders in female refugees as early as possible. Our findings show that the following factors are associated with symptoms of depression and anxiety: level of education, country of origin, and number of PTEs. Currently, many female refugees from Ukraine are arriving in Germany. Even though their country of origin is different from that of our sample, our findings can help estimate the prevalence of symptoms of depression and anxiety and associated factors in female Ukrainian refugees. Psychological support and psychotherapy must be made available as a low-threshold service for female refugees who need it. This is an important step in the integration of female refugees into their host countries. In addition, female refugees arriving in Germany should be screened for symptoms of mental disorders. The refugee population is always changing; therefore, ongoing research is needed to assess the mental health of female refugees in Germany.

## Data availability statement

The raw data supporting the conclusions of this article will be made available by the authors, without undue reservation.

## Ethics statement

The studies involving humans were approved by Ethics Committee of the Rhineland-Palatinate Medical Association. The study is registered under the number 837.316.16 (10635). The studies were conducted in accordance with the local legislation and institutional requirements. The participants provided their written informed consent to participate in this study.

## Author contributions

HB: Writing – original draft. RK: Writing – review & editing. PK: Writing – review & editing. PD: Writing – review & editing. ClK: Writing – review & editing. SL: Writing – review & editing. ChK: Writing – review & editing. JJ: Writing – review & editing. MS-O: Writing – review & editing. UZ: Writing – review & editing.

## Footnotes

^1^Protection seekers are people applying for asylum, people with asylum, and refugees in Germany.

^2^Since February 24, 2023, with the introduction of martial law, most Ukrainian men of draft age were banned from leaving Ukraine.
